# Six additional mitochondrial genomes for North American nightsnakes (Dipsadidae: *Hypsiglena*) and a novel gene feature for advanced snakes

**DOI:** 10.1080/23802359.2020.1797573

**Published:** 2020-07-29

**Authors:** Edward A. Myers, Daniel G. Mulcahy

**Affiliations:** a Department of Vertebrate Zoology, National Museum of Natural History, Smithsonian Institution, Washington, DC, USA; b Global Genome Initiative, National Museum of Natural History, Smithsonian Institution, Washington, DC, USA

**Keywords:** Hypsiglena, nightsnake, Squamata, Dipsadinae, mtDNA

## Abstract

The North American nightsnakes in the genus *Hypsiglena* is composed of nine named and at least two unnamed species. Here, we provide the first mt-genome of *H. affinis*, an additional mt-genome for *H.* sp. nov. 1, and four additional mt-genomes from the widespread *H. jani*. These mtDNA genomes were sequenced using both Illumina and Ion Torrent sequencing technologies. The resulting genomes contained the expected 13 protein coding genes, 22 tRNA genes, 2 rRNA genes, and 2 control regions typical of colubroid snakes. Two of the *H*. *jani* samples had partial tRNA^Ile^ genes upstream of CR2 which has not been previously documented in colubroid snakes. A maximum likelihood gene-tree based on these data combined with previously published sequence data recovers a well-supported phylogeny and is in concordance with previous estimates of evolutionary relationships in this group.

North American nightsnakes are small, nocturnal colubroid snakes widespread across the arid Nearctic (Mulcahy 2008). This genus contains nine known species, and two unnamed species awaiting formal recognition (Mulcahy 2008; Mulcahy et al. 2014). Mitochondrial genomes for nearly all species have previously been sequenced to better understand this group’s historical biogeography (Mulcahy and Macey 2009). Here we sequence four additional mitochondrial genomes from *Hypsiglena jani* Dugès, 1865, one *H. affinis* Boulenger, 1894, and one of an unnamed species.

Specimens/tissues of *H. jani* were collected from Hidalgo county, New Mexico, USA (AMNH R-504522: 31.897, −109.216; AMNH R-504524: 32.028, −109.035), Cadereyta de Montes, Queretaro, Mexico (AMNH R-504774: 20.925, −99.756), and Tlahualilo, Durango, Mexico (AMNH R-504773: 26.696, −103.747). The *H. affinis* tissue voucher is from Jalisco, Mexico (LSUMZ 39533: 19.985, −103.630) and the ‘*H.* sp. nov. 1′ was collected from Cochise County, Arizona, USA (AMNH R-504527: 32.210, −108.951). The *H. affinis* was processed as in Mulcahy et al. (2014). For the others, DNA was extracted using Qiagen DNeasy Tissue Kits (Valencia, CA), genomic libraries were prepared using the Nextera XT kit, shearing DNA fragments to an average base pair length of 480, and were sequenced on an Illumina MiSeq with paired-end 250 base pair reads (Illumina, San Diego, CA). Sequences were initially mapped to a *H. jani* mitochondrial genome (EU728592) in Geneious v10.2.6 (Biomatters Ltd, 2005–2017). After constructing a consensus sequence, all reads were mapped back to this consensus to construct the final assembly. All six newly assembled mitochondrial genomes contained 13 protein coding genes, 22 tRNA genes, 2 rRNA genes, and 2 control regions which is typical of colubroid snakes (Kumazawa et al. 1998). The control regions were difficult to assemble in C-rich regions of several samples, unobtainable in AMNH R-504774 and short three C’s in AMNH R-504527, likely due to the difficulties of sequencing through homopolymers using Illumina sequencing. The recovered genome lengths and corresponding GenBank numbers of each specimen are: AMNH R-504522 (MT561495) 17,200; AMNH R-504524 (MT561496) 17,235; AMNH R-504527 (MT561497) 17,235; AMNH R-504774 (MT561500) 17,205; AMNH R-504773 (MT561498) 17,202; LSUMZ 39533 (MT561499) 17,190 base pairs. Mean depth of coverage ranged from 23.6–53.6x (average: 37.9). Two mt-genomes (AMNH R-504524 and AMNH R-504527) contained identical putative pseudogenes, partial tRNA^Ile^, on the 5′ end of the second control region.

Our six new mt-genomes were aligned with data from 14 published *Hypsiglena* mt-genomes, ∼5kb of mtDNA data from *H. tanzeri*, and complete mt-genomes of *Pseudoleptodeira latifaciata*, *Sibon nebulatus*, *Imantodes cenchoa*, *Leptodeira septentrionalis* were included as outgroups (Mulcahy 2008; Mulcahy and Macey 2009; Mulcahy et al. 2014). A maximum likelihood phylogeny was generated with RAxML v8.2.3 using the simultaneous rapid-bootstrap (1000 replicates) and thorough ML search (Stamatakis 2014), with each gene in a separate partition and only one control region (CR2), with the GTRCAT substitution model. The phylogeny (Figure 1) is well supported and in concordance with previous estimates of relationships within the genus *Hypsiglena* (Myers et al. 2013, 2017; Mulcahy et al. 2014).

**Figure 1.**
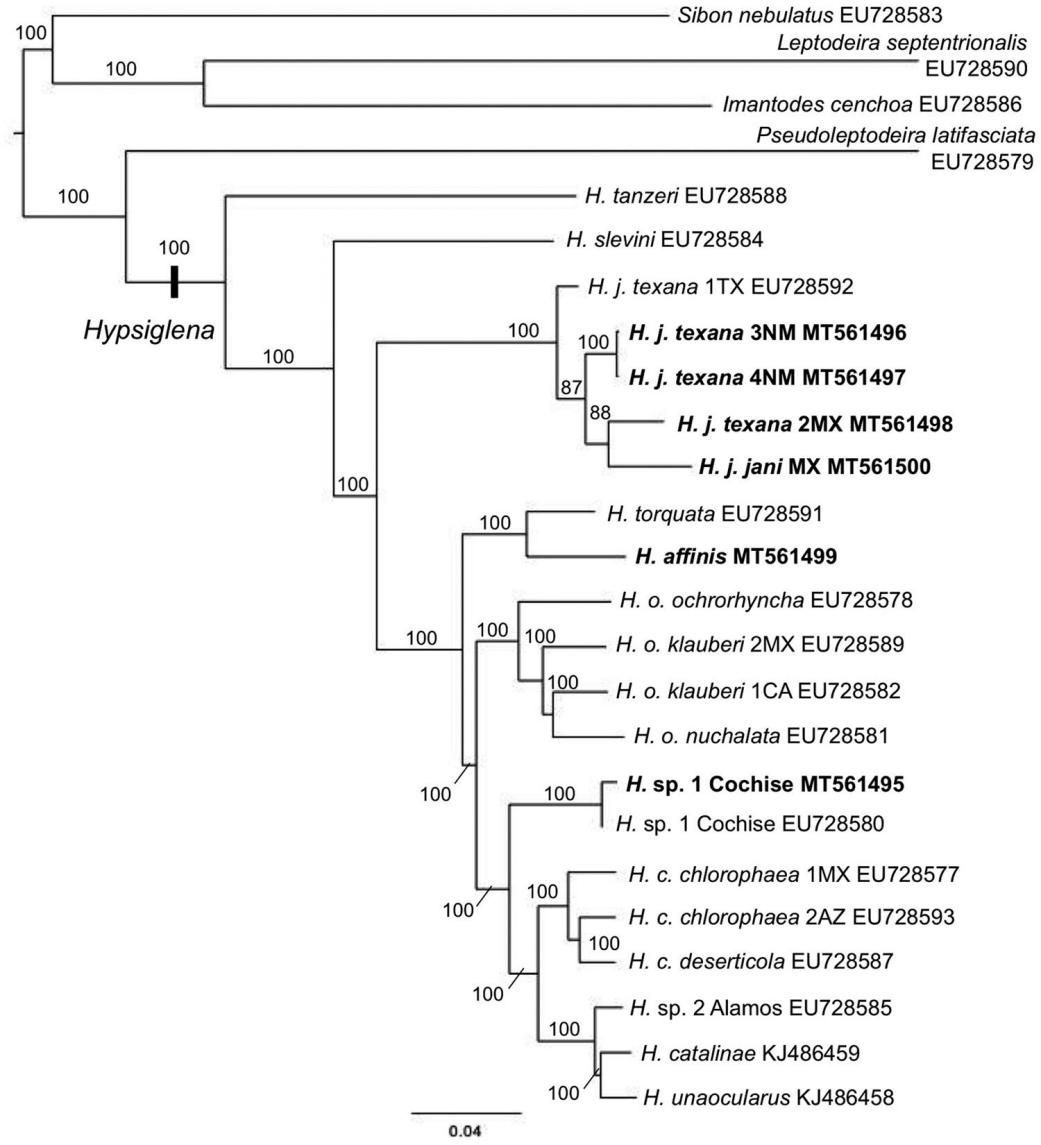
Maximum likelihood gene-tree based on complete mt-genomes of nightsnakes (*Hypsiglena*). Bootstrap values of support (based on 1000 replicates) are shown for each node. *H. tanzeri* is represented by ∼5kb of mtDNA data. New mt-genomes are shown in bold font.

A previous study of nightsnakes produced 12 complete mt-genomes (Mulcahy and Macey 2009), with four from each of *H. ochrorhyncha* and *H. chlorophaea*. The other widespread species *H. jani*, as well as *H. slevini*, *H. torquata*, and the undescribed *H*. sp. nov. 1, were represented by single individuals. A later study (Mulcahy et al. 2014) added two mt-genomes to the southern portion of *H. ochrorhyncha*, elevating two species (*H. catalinae* and *H. unaocularus*) and identified another undescribed species from Sonora, MX (*H*. sp. nov. 2). All *Hypsiglena* mt-genomes to-date showed no deviations in gene-order from previously published Dipsadines (Mulcahy and Macey 2009; Sun 2017), with the exception of lacking the partial tRNA^Pro^. Here, we provide the first mt-genome of *H. affinis*, an additional one for *H.* sp. nov. 1, and four additional mt-genomes from the widespread *H. jani*. Two individuals (AMNH R-504524 and AMNH R-504524) near the contact zone between *H. jani* and *H.* sp. nov. 1, show partial tRNA^Ile^ genes in the same region as the pseudo tRNA^Pro^ reported in *Dinodon* (NC 001945; Kumazawa et al. 1998). Both pseudo-genes, and their respective parent genes (tRNA^Pro^ and tRNA^Ile^), reside upstream of control regions. In *Dinodon*, the pseudo-tRNA^Pro^ was found upstream of CR2, with the functional tRNA^Pro^ upstream of CR1 (Kumazawa et al. 1998), as in most vertebrates. Viperids show two unique gene-orders in these regions, where both have functional tRNA^Pro^ upstream of CR2 and one has a pseudo tRNA^Pro^ upstream of CR1; the other lacks the pseudo tRNA^Pro^ (Yan et al. 2008). The presence of two CRs in advanced snakes has been attributed to tandem duplication during replication or concerted evolution (Kumazawa et al. 1998; Dong and Kumazawa 2005). The repetitive nature of tRNAs bordering duplicate control regions are likely involved in, or are a product of, the evolutionary events driving this phenomenon.

## Data Availability

All Illumina and Ion Torrent reads have been accessioned on the NCBI Short Read Archive with BioProject ID PRJNA636782 (https://www.ncbi.nlm.nih.gov/bioproject/PRJNA636782/). Assembled and annotated mt-genomes are accessioned on GenBank under the following accession numbers MT561495-MT561500 (https://www.ncbi.nlm.nih.gov/nuccore/MT561495; https://www.ncbi.nlm.nih.gov/nuccore/MT561496; https://www.ncbi.nlm.nih.gov/nuccore/MT561497; https://www.ncbi.nlm.nih.gov/nuccore/MT561498; https://www.ncbi.nlm.nih.gov/nuccore/MT561499; https://www.ncbi.nlm.nih.gov/nuccore/MT561500).

## References

[CIT0001] Dong S, Kumazawa Y. 2005. Complete mitochondrial DNA sequences of six snakes: phylogenetic relationships and molecular evolution of genomic features. J Mol Evol. 61(1):12–22. 16007493 10.1007/s00239-004-0190-9

[CIT0002] Kumazawa Y, Ota H, Nishida M, Ozawa T. 1998. The complete nucleotide sequence of a snake (*Dinodon semicarinatus*) mitochondrial genome with two identical control regions. Genetics. 150(1):313–329. 9725849 10.1093/genetics/150.1.313 PMC1460336

[CIT0003] Mulcahy DG. 2008. Phylogeography and species boundaries of the western North American Nightsnake (*Hypsiglena torquata*): revisiting the subspecies concept. Mol Phylogenet Evol. 46(3):1095–1115. 18226930 10.1016/j.ympev.2007.12.012

[CIT0004] Mulcahy DG, Macey JR. 2009. Vicariance and dispersal form a ring distribution in nightsnakes around the Gulf of California. Mol Phylogenet Evol. 53(2):537–546. 19501659 10.1016/j.ympev.2009.05.037

[CIT0005] Mulcahy DG, Martínez-Gómez JE, Aguirre-León G, Cervantes-Pasqualli JA, Zug GR. 2014. Rediscovery of an endemic vertebrate from the remote Islas Revillagigedo in the Eastern Pacific Ocean: the Clarión Nightsnake lost and found. PLoS One. 9(5):e97682. 24837300 10.1371/journal.pone.0097682 PMC4023976

[CIT0006] Myers EA, Hickerson MJ, Burbrink FT. 2017. Asynchronous diversification of snakes in the North American warm deserts. J Biogeogr. 44(2):461–474.

[CIT0007] Myers EA, Weaver RE, Alamillo H. 2013. Population stability of the northern desert nightsnake (*Hypsiglena chlorophaea deserticola*) during the Pleistocene. J. Herpetol. 47(3):432–439.

[CIT0008] Stamatakis A. 2014. RAxML version 8: a tool for phylogenetic analysis and post-analysis of large phylogenies. Bioinformatics. 30(9):1312–1313. 24451623 10.1093/bioinformatics/btu033 PMC3998144

[CIT0009] Sun F-H. 2017. Revealing the complete mitochondrial genome of *Thermophis baileyi* Wall, 1907 (Reptilia: Colubridae) through the Next-Generation Sequencing. Mitochondrial DNA Part B. 2(2):391–392. 33473837 10.1080/23802359.2017.1347902 PMC7799630

[CIT0010] Yan J, Li H, Zhou K. 2008. Evolution of the mitochondrial genome in snakes: gene rearrangements and phylogenetic relationships. BMC Genomics. 9:569 19038056 10.1186/1471-2164-9-569 PMC2632649

